# Unilateral biportal endoscopic spinal surgery for the treatment of Bertolotti syndrome in an adolescent, a case report

**DOI:** 10.3389/fsurg.2026.1825794

**Published:** 2026-07-21

**Authors:** Lingfeng Ren, Tianhua Qiao, Yan Zhang, Yong Wang, Xin Zhang

**Affiliations:** Second Department of Spinal Surgery, Central Hospital Affiliated to Shenyang Medical College, Shenyang, China

**Keywords:** BS, LSTV, minimally invasive spinal surgery, teenagers, UBE

## Abstract

**Background:**

Bertolotti syndrome (BS), associated with lumbosacral transitional vertebrae (LSTVs), is characterized by back pain and/or radicular symptoms due to the compression of the fifth lumbar (L5) exiting nerve roots. The surgical treatment of BS by partial resection of L5 transverse process (TP) or sacral alar via diverse techniques achieves encouraging clinical results. However, related reports on endoscopic surgery for adolescent patients with BS are rare. We here present a case of a 17-year young girl with BS who underwent endoscopic resection of left side L5/S1 pseudoarthrosis by using a unilateral biportal endoscopic spinal surgery (UBESS, UBE) technique.

**Methods:**

The current study presented a 17-year-old high-school girl who suffered from 4 months persistent lower back pain (LBP) at her first visit to our hospital. After 3 months conservative treatment, the young girl presented a severe back pain combined with left radicular pain for 1.5 months. Preoperative imaging revealed an obviously LSTV (type of Castellvi IIa) and a left side L5/S1 pseudoarthrosis. After carefully preoperative preparation and a diagnostic anesthetic block, the patient accepted an endoscopic decompression surgery via a UBE technique. During the surgery, the left hypertrophic L5 TP as well as the sacral alar were partially resected and the L5 nerve root was decompressed.

**Results:**

Under the intraoperative endoscopic view, the left L5/S1 pseudoarthrosis was removed by using a high-speed bur. And the left L5 nerve root was perfectly decompressed without vice injury. The lower back pain and radicular pain were greatly relieved on the next postoperative day after the minimally invasive spinal surgery. The visual analogue scale (VAS) back pain score was improved from 8 points preoperative to 2 points postoperative, while the leg pain score was improved from 8 points preoperative to 1 points postoperative. The symptoms of back pain and radiculopathy were thoroughly vanished at the 3-month follow-up. And the patient never returned back to our clinic for further medical consultation.

**Conclusion:**

Endoscopic decompression via an UBE technique can be an effective choice for adolescent patients with BS after failed conservative therapy.

## Introduction

BS, due to a congenital spinal deformity of LSTV in the lumbosacral region, is defined as a combination of LBP and L5 radiculopathy (1). As an important contributor of LBP, the prevalence of BS varies from 4% to 8% in adult population (2). It was reported that the incidence of BS in young population (under-30 age) is 11.4%, which is higher than the overall incidence of 4.6% according to a MRI-based consecutive study of 769 patients (3). Since no consensus or guidelines on the treatment of BS, diverse treatments including nonsurgical methods like NSAIDs, epidural steroid injection, physical therapy as well as surgical measures like nerve ablation and processectomy or L5/S1 fusion were reported (4). As to the surgical treatment of BS in adolescents, related reports are even rare (5, 6).

For surgical interventions of patients with BS, diverse procedures including resection of anomalous TP/body spur and pseudoarticulation or even L5/S1 fusion via different techniques were applied (Table 1). However, although these treatment methods can help alleviate symptoms, their efficacy and safety still have significant limitations. Arthrodesis or spondylotomicectomy may relieve local mechanical pain initially, but long-term benefit is often limited. Although spinal fusion provides more stable reconstruction, treatment of LSTV-related lesions—particularly with long-segment fusion—still carries risks of pseudoarticulation formation and revision surgery (7, 8). Recently, endoscopic spinal surgery also achieved encouraging clinical effect in the treatment of BS. Byapak Paudel reported a study of successfully decompression in treating of 3 patients with BS by a percutaneous uni-portal full endoscopic technique (9). Endoscopic resection of pseudoarticulation by a similar technique were also reported by other researchers (10, 11).

**Table 1:** Diverse approach and surgical procedures in treatment of BS.

Approach	Technique	Surgical procedure	Year	Authors	Follow-up	Outcomes
Anterior						
		Extraforaminal decompression of the left L5 transverse process and bony spur through extraperitoneal anterior approach	1997	Abe, E et al	1 year	Returned to job without LBP or numbness
		Decompression and resection of osteophyte and bony spur through anterior extraperitoneal approach (Fraser incision)	2013	Kikuchi, Kazuma et al.	1 year	LBP and leg pain relieved and functionally improved
		L5/S1 pseudoarthroses resection via anterior retroperitoneal approach	2013	Malham, Gregory M et al	2 year	Long term symptomatic relief
Posterior						
	Open/mini-open					
		Resection of the transverse process	1989	Jönsson, B et al	6–42 months	7 patients reported total alleviation of pain 2 patients had unchanged symptoms
		Resection of anomalous transverse processes utilizing intraoperative three-dimensional image-guided navigation	2017	Babu, Harish et al	6 months 9 months	Resolution of pain
		Pure L5 transverse processectomy	2017	Ju, Chang Il et al	6.5 months	12 patients showed excellent results 41 patients showed good results 6 patients showed fair results 2 patients showed poor results
		Resection of the left transverse Mega-apophysis with Wiltse approach	2019	Cuenca, C et al	1 year	Complete disappearance of pain
		Resection of the right L6 transverse process	2001	Brault, J S et al	1 year	Resume a normal lifestyle without limitations
		Decompression by resection of the bony spur using posterior approach	2004	Ichihara, Kazuhiko et al	2 years	No longer pain in hip and leg and had only occasional LBP
		Resect the anomalous transverse process and the accompanying pseudoarticulation via minimally invasive approach	2008	Ugokwe, Kene T et al	6 months	Significant (90%) improvement in LBP and left lower extremity pain
		Nerve root decompression via posterior approach	2010	Weber, Jochen, and Ralf-Ingo Ernestus	1 year	No radicular or lumbar pain
		Posterolateral fusion and resection of the transitional articulation	1993	Santavirta, S et al	2–17 years	10 patients improvement 7 patients had no low back pain at all 11 of 13 patients still had episodes of sciatica 5 patients had functionally normal mobility of the lower back 8 patients had poor mobility
		Posterolateral fusion	2018	Adams, Ryan et al	2 weeks	Complete resolution of the pain symptoms
	Endoscopic/Microscopic technique					
		Endoscopic resection of the left pseudoarticulation	2023	Stein, Eric et al	n/a	Sustained improvement in pain
		Endoscopic partial transverse process and sacral alar resection	2024	Tatsumura, Masaki et al	2 years	Resolution of pain
		Full-Endoscopic J-Shaped transforaminal L5 nerve decompression	2024	Chang-Il Ju et al	n/a	Less invasive, faster recovery, less blood loss
		Full endoscopic resection of the anomalous transverse process	2025	Kawasaki, Toshinari et al	3 months	LBP dramatically resolved
		Extraforaminal decompression of right L6 via METRx system	2011	Shibayama, M et al	30 months	Able to walk well and return to work
		Posterior decompression by resection of the osteophytes using operating microscope	2011	Miyoshi, Yasuyuki et al	1 year	Free of symptoms
		Resection of the LSTV by minimally invasive, paramedian tubular-based approach	2014	Li, Yumeng et al	4 years	3 patients complete resolution of LBP 2 patients reduce LBP 2 patients initial relief but return of LBP at 1 and 4 years postoperatively
		Discectomy at L4-5 and resection of the left L5 transverse process using Minimally invasive microendoscopic resection	2014	Takata, Yoichiro et al	n/a	Improve in LBP and sciatic pain
		Minimally invasive “wide” L5 transverse process resection	2021	Kawtharani, Sarah et al	n/a	Full resolution of pain and radiculopathy
		Posterior resection using intraoperative three-dimensional (3D) navigation through microendoscopic	2021	Shinonara, Kensuke et al	2 months	free from the left low back pain
		Minimally invasive microscopic tubular articular resection	2022	Chang, Chun-Jen et al	2 years	No interval changes or re-articulation
		Mini-open tubular microsurgical transverse processectomy	2022	Afana, Hatem et al	3–36 months	Alleviation of pain obviously

To date, UBE technique is extensively applied in most types of spinal disorders including cervical spondylotic radiculopathy, Lumbar degenerative disc herniation, canal stenosis, infection and tumor (12–18) In the present study, we reported the clinical outcome of UBE technique in treating of a 17-year adolescent with BS.

## Case presentation

**Figure 1:**
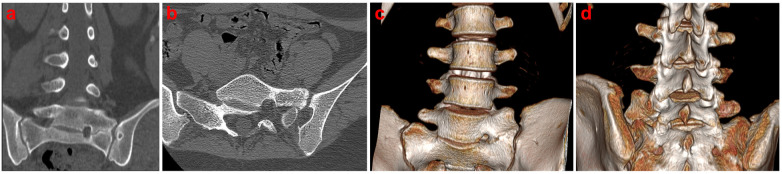
Preoperative sagittal **(a)** and axial **(b)** CT scans of the lumbar region, as well as the three-dimensional computed tomographic of the lumbar spine **(c,d)**, revealed that the left transverse process of L5 was enlarged and formed an incomplete bony fusion with the sacrum through fibrous joint spaces.

A 17-year-old female high-school student had come to the spine surgery outpatient of the Central Hospital Affiliated to Shenyang Medical College with the complaint of persistent LBP lasting for 4 months. The three-dimensional computed tomographic (3D-CT) scans indicated an obviously LSTV (Castellvi IIa) with the character of a left hypertrophic-L5 TP and a formatted pseudoarticulation with the sacrum (Figures 1a–d). The patient accepted a conservative treatment including non-steroidal anti-inflammatory drugs (NSAIDs) and physical exercise primarily. However, two-months later, the young patient came back for further help due to the even worse back pain combined with left sciatica for 1.5 months.

**Figure 2:**
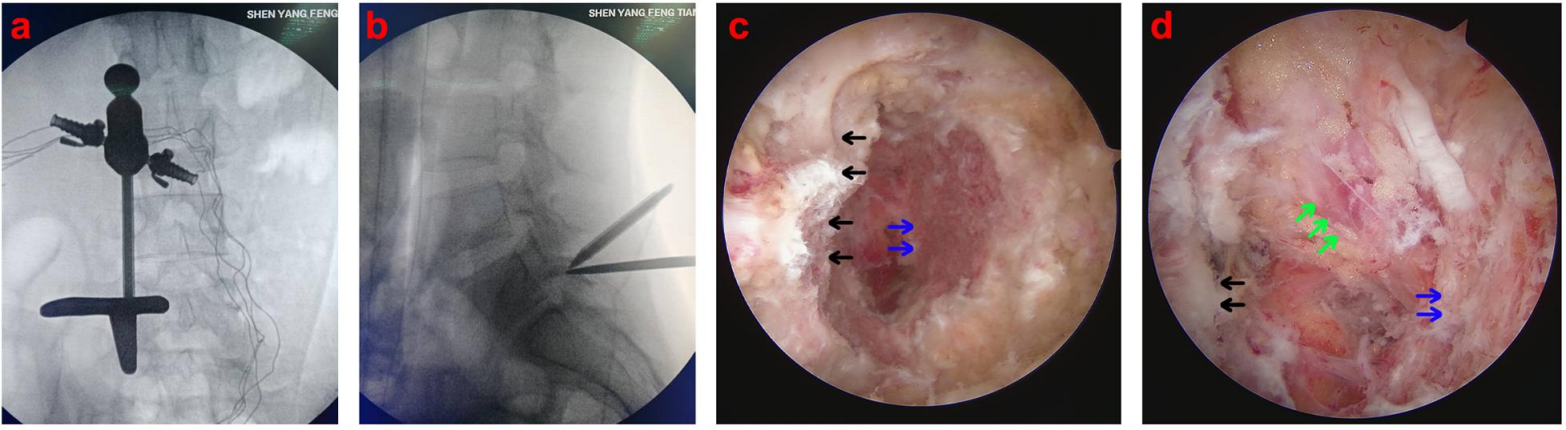
Intraoperative anteroposterior **(a)** and lateral **(b)** X-rays confirmed that the channel was located at the isthmus. L5 TP (black arrow) and the sacrum ala (blue arrow) was partially removed **(c)**, and the compression on the left L5 nerve root (green arrow) has been relieved **(d)**.

A diagnostic anesthetic block was performed initially. An immediate alleviated LBP was admitted by the young patient. Then, an endoscopic spinal surgery was performed for the patient via a UBE technique in the prone position under general anesthesia (Figures 2a,b). At the L5/S1 level, two approximately 1.5-cm horizontal incisions were made cranial and caudal to the left L5 transverse process, each about 1.5 cm from the target site. After sequential incision of the skin, subcutaneous tissue, and deep fascia, the surrounding soft tissue was dissected to expose the hypertrophic left L5 transverse process and its pseudoarticulation with the sacrum. The endoscope and working instruments were then introduced, and fluoroscopy was repeated to confirm the operative level. A low-temperature plasma radiofrequency probe was used to remove the dorsal soft tissue overlying the pseudoarticulation. Under the endoscope, a high-speed bur was used to partially resect the hypertrophic L5 TP and the sacrum ala (Figure 2c). The left L5 nerve root was identified and freely released (Figure 2d). During the operation, pay close attention to protecting the L5 nerve root and strictly control the range of bone resection to ensure that the artificial joint is separated sufficiently while avoiding excessive bone removal.

**Figure 3:**
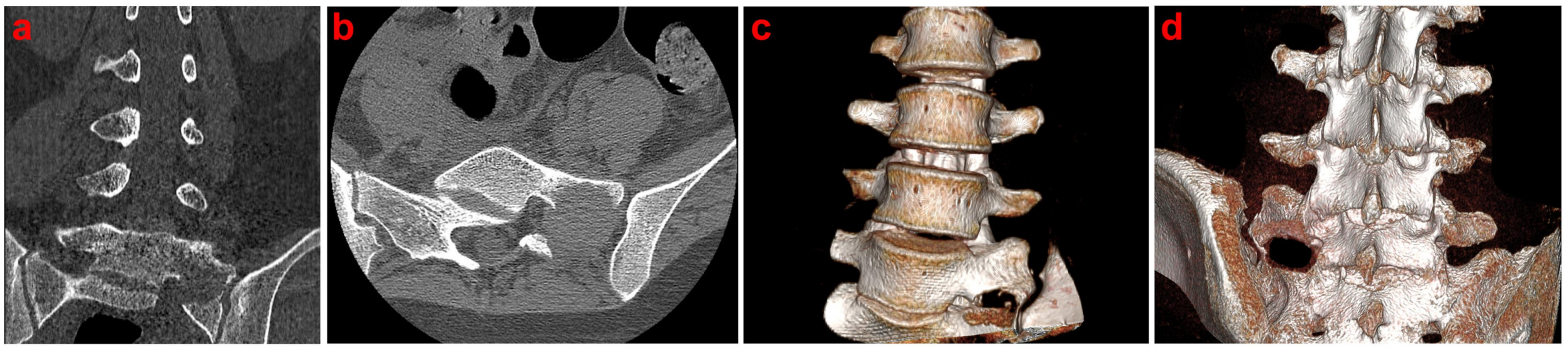
Postoperative sagittal **(a)** and axial **(b)** CT scans of the lumbar region, as well as three-dimensional computed tomography (CT) scans of the lumbar spine **(c,d)**, show that the artificial joint has been removed.

The lower back pain and left radicular pain were greatly relieved on the next postoperative day after the UBE surgery. The VAS back pain score was improved from 8 point preoperative to 2 point postoperative, while the VAS leg pain score was improved from 8 point preoperative to 1 point postoperative. The post-operative 3D-CT scans showed that the formatted pseudoarticulation between hypertrophic L5 TP and sacrum ala was completely removed (Figures 3a–d). The symptoms of back pain and radiculopathy were thoroughly vanished at the 3-month follow-up. And the patient never returned back to our hospital for further medical consultation.

## Discussion

Due to the biological basis of abnormality, BS presents diverse chronic LBPs like pain in the sacroiliac joint, groin, and hip region, and may or not combine with radiculopathy (4). To date, no guidelines or consensus systematically focused on the treatment of BS. In 2024, Ahish Chitneni and workmates proposed a treatment algorithm for patients with LBP secondary to BS (19). The treatment of BS depends on the severity of the clinical symptoms and the classification of LSTV (20). For symptomatic BS with type II of LSTV, classified by direct bone contact between the L5 TP and the ala of the sacrum, surgical methods like nerve ablation, surgical fusion, or steroid injections directly into the pseudoarticulation were usually applied (4, 20). The current young patient presented a Castellvi IIa of LSTV, the unilateral pseudoarticulation undergoes repeated mechanical stress during daily activities, potentially causing degenerative and reparative changes, including fibrosis, chondrocyte clustering, and focal necrosis, and may directly generate pain. Therefore, anti-inflammatory treatment with nonsteroidal anti-inflammatory drugs is often effective. However, because of the worsening low back pain and left-sided sciatica and the patient's young age, endoscopic resection was selected after a preoperative diagnostic anesthetic block confirmed the pain source (21). A preoperative diagnostic anesthetic block greatly improved her symptom, then a further endoscopic spinal surgery was performed.

As a type of widely recognized and rapidly developing novel minimally invasive spinal surgery, UBE technique achieved encouraging preliminary clinical results in treatment of types of spinal disorders like disc herniation, canal stenosis, ossification of the ligamentum flavum (OLF), and intracanal tumors (13, 22–29) Previous literature reports indicate that compared with traditional open surgery and microscopic surgery, patients accepted UBE technique benefit from shorter hospital stays, lower blood loss, comparable complication rates and faster recovery time (22, 30). However, UBE is associated with a relatively steep learning curve, and in complex surgical settings, technical manipulation and instrumentation may increase the risk of dural injury (31). If the intraoperative water pressure is too high, it may lead to symptomatic Postoperative Spinal Epidural Hematoma, but its occurrence rate is relatively low (32). UBE is not suitable for all patients either. For instance, high-grade spondylolisthesis, deformity correction, fractures, and pathologic conditions, the use of purely endoscopic techniques may not yield satisfactory results (33). Therefore, careful selection of patients remains crucial. Compared with previous reports of UBE for BS, the patient in the present case was younger, had less severe disc degeneration and better segmental stability, and showed more rapid early postoperative recovery with greater neurological improvement. In addition, the long-term outcome of this case may provide further follow-up value (34). Meanwhile, compared with other cases of adolescent lumbar discectomy for lumbar disc herniation, this case should be understood as demonstrating the feasibility of the UBE technique in a specific patient population (35). As to the current case, the next day after the UBE surgery, the patient achieved a great improvement of both LBP and leg pain. The 3-month follow-up also present a satisfying clinical outcome.

In conclusion, UBE technique provides a safe, effective and minimally invasive surgical alternative for treatment symptomatic BS with failed conservative treatment.

## Data Availability

The original contributions presented in the study are included in the article/Supplementary Material, further inquiries can be directed to the corresponding author.
